# Marine Microbial Diversity as Source of Bioactive Compounds

**DOI:** 10.3390/md20050304

**Published:** 2022-04-29

**Authors:** Khaled A. Shaaban

**Affiliations:** Center for Pharmaceutical Research and Innovation, Department of Pharmaceutical Sciences, College of Pharmacy, University of Kentucky, Lexington, KY 40536, USA; khaled_shaaban@uky.edu

Natural products continue to be a major inspiration and untapped resource for bioactive drug leads/probes. Natural products (NPs) or biologically inspired NPs account for approximately 24% of currently approved drugs [1]. Concerning the discovery and development of anticancer and antibiotics, microbially produced candidates now account for a very high proportion of drugs that are commonly prescribed. Compared to other natural sources, marine microbial diversity has now become a potential source for drug lead discovery. Oceans and seas cover more than 70% of the earth’s surface and are massively complex, containing diverse assemblages of life forms. Marine bacteria, fungi and other microorganisms develop unique metabolic and physiological capabilities that enable them to survive in extreme habitats and to produce compounds that might not be produced by their terrestrial counterparts. In the last few decades, the systematic investigation of marine/marine-derived microorganisms as sources of novel biologically active agents has exponentially increased. Overall, this Special Issue contains eight articles, including six research articles on different topics related to the microbial natural products derived from marine microbes and two comprehensive review articles. In the following sections, we provide a brief overview of what the reader will find in this Special Issue.

Shaaban et al. reported the isolation and structural identification of three new isoquinolinequinone derivatives (mansouramycins E–G) in addition to the previously reported known compounds mansouramycins A and D from the ethyl acetate extract of the marine-derived *Streptomyces* sp. isolate B1848. The chemical structures of these compounds were elucidated by NMR (1D, 2D), HRMS, comparison with related compounds and computer-assisted methods. The cytotoxic activity of the isolated mansouramycins has been evaluated in a panel of up to 36 tumor cell lines, indicating significant cytotoxicity and good tumor selectivity for the new isolated compound mansouramycin F [2].

Four new cytotoxic indole-diterpenoids (penerpenes K-N), along with twelve other known compounds, have been discovered by Dai et al. from the fermentation broth produced by adding L-tryptophan to the culture medium of *Penicillium* sp. KFD28. The structures of the new compounds were elucidated extensively by NMR, HRMS data analyses and ECD calculations. Penerpene N represents the second example of paxilline-type indole diterpene bearing a 1,3-dioxepane ring. Three compounds (penerpene N, epipaxilline, emindole SB) were found to be cytotoxic to cancer cell lines, of which the known compound, epipaxilline, was the most active and showed cytotoxic activity against the human liver cancer cell line BeL-7402 with an IC_50_ value of 5.3 μM. Moreover, six compounds, namely paxilline, 7-hydroxyl-13-dehydroxypaxilline, 7-hydroxypaxilline-13-ene, 4a-demethylpaspaline-4a-carboxylic acid, PC-M6 and emindole SB, showed antibacterial activities against *Staphylococcus aureus* ATCC 6538 and *Bacillus subtilis* ATCC 6633 [3].

Shu et al. discovered viridicatol as a lead derivative for allergic diseases treatments. Viridicatol is a new quinoline alkaloid derivative purified from the deep-sea-derived fungus *Penicillium griseofulvum*. The structure of viridicatol was established by NMR and X-ray diffraction analysis. In the in vivo biological investigation of allergic reaction treatments in a mouse model study, viridicatol was found to amilorate the inflammatory mediator, stabalized the mast cell elivation of anaphylaxis and repaired the intestinal barrier in mice by suppressing mast cell activation [4].

The study by Lu et al. explored an example of how new strategies could accelerate the discovery of new antibiotics from highly productive natural sources. This article focused on an integrated strategy of combining phylogenetic data and bioactivity tests with a metabolomics-based dereplication approach to fast track the selection process of Mangrove actinomycetia for the discovery of novel biologically active natural products. Metabolomics technologies have been utilized to considerably aid traditional antibiotic discovery approaches in strain prioritization, resulting in increased efficiency in the discovery of new antibiotics from these highly productive and diverse ecosystems. It is a great example of using non-traditional bioactivity and/or taxonomy-based dereplication methods for the selection of new microbial strains for future natural product discoveries. In this study, a total of 521 actinomycetial strains affiliated to 40 genera in 23 families were isolated from 13 different mangrove soil samples by a culture-dependent method. A total of 179 strains affiliated to 40 different genera with unique colony morphology were selected to evaluate antibacterial activity against 12 indicator bacteria. Out of the 179 tested isolates, 47 showed activity against at least one of the tested pathogens. An analysis of 23 out of 47 active isolates using UPLC-HRMS-PCA revealed 6 outliers. Further analysis using the OPLS-DA model identified five compounds from two outliers contributing to bioactivities against drug-sensitive *A. baumannii*. Two *Streptomyces* strains (M22 and H37) were rapidly prioritized for producing potentially new compounds. The scale-up fermentation of *Streptomyces* sp. M22 afforded two new trioxacarcins with keto-reduced trioxacarcinose B, gutingimycin B and trioxacarcin G, together with the known gutingimycin [5].

SARS-CoV-2 (severe acute respiratory syndrome coronavirus-2) is a novel coronavirus strain that emerged at the end of 2019 and has resulted in millions of deaths so far. Marine sulfated polysaccharides (MSPs) are a group of natural products that have been reported from a variety of marine sources. They have recently gained significant attention and are widely examined against a variety of viral diseases. Salih et al. presented a comprehensive report and modeling analysis on marine sulfated polysaccharides from different marine sources as potential antiviral medicines, with an emphasis on SARS CoV-2. They aimed to compile a thorough report on MSPs and their antiviral activities against diverse virus species based on studies published over the last 25 years. The reported MSPs were subjected to molecular docking and dynamic simulation experiments, and it was found that nine of the investigated MSPs candidates exhibited promising results, taking into consideration the newly emerged SARS CoV-2 variants, of which five were not previously reported to exert antiviral activity against SARS CoV-2, including sulfated galactofucan, sulfated polymannuroguluronate (SPMG), sulfated mannan, sulfated heterorhamnan and chondroitin sulfate E (CS-E). These promising results shed light on the importance of sulfated polysaccharides as potential SARS-CoV-2 inhibitors [6].

An original article by Ben Hlima et al. focused on the discovery of new-lipolytic enzymes of biotechnological interest from microalgae with the aid of genomic mining by combining bioinformatics analysis and functional screening to find novel lipases biocatalysts. The in silico characterization of 14 putative *Chlorella vulagaris* lipases with different cellular localizations has been reported in the current study. Membrane-associated lipases were also detected and described in the article for the first time in this species. The 14 lipases display an acyl hydrolase motif (GXSXG) and belong to the α/β hydrolase lipase 3 family and the GX class. These putative lipases could be potential candidates for metabolic engineering to improve microalgae lipid productivity. Finally, the authors of this manuscript have also reported, for the first time, a putative lysosomal acid lipase produced by a green microalgae [7].

Lei Chen et al. provided a comprehensive review of natural products from microorganisms associated with sea cucumbers. Sea cucumbers are a class of marine invertebrates that are extensively used as a source of food in Asian cuisines and have reported pharmacological activities. Numerous microorganisms have been associated with sea cucumbers. Seventy-eight genera of bacteria belonging to forty-seven families in four phyla and twenty-nine genera of fungi belonging to twenty four families in the phylum Ascomycota have been cultured from sea cucumbers. Sea-cucumber-associated microorganisms produce diverse secondary metabolites with various biological activities, including cytotoxic, antimicrobial, enzyme-inhibiting and antiangiogenic activities. In this review, the authors have summarized the list of 145 natural products isolated from microorganisms associated with sea cucumbers between 2000 and 2021, which include polyketides, alkaloids and terpenoids as well as their reported biological activities [8].

Co-cultivation is one of the strategies used for drug discovery and has been known as an effective approach for the enhancement of the production of natural products from microorganisms. Jianwei Chen at al. provided a comprehensive review on the structural diversity of marine microbial metabolites based on the co-culture strategy. As reported by the authors, co-culturing of two or more marine microorganisms together in a solid or liquid medium in a certain environment can activate silent biosynthetic genes to produce cryptic natural products that do not exist in monocultures of the partner microbes based on either their competition or synergetic relationship. This review article by Chen et al. focuses on the significant and excellent examples covering sources, types, structures and the bioactivities of secondary metabolites based on the co-cultures of marine-derived microorganisms from 2009 to 2019. They have summarized 154 phytomolecules reported to be produced by the marine microorganism co-culture with examples of novel and bioactive natural products. They have also provided a detailed discussion on the prospects and current challenges in the field of co-culture approaches [9].

In summary, the research articles on the topic presented in this Special Issue reveal the potential of marine microorganisms and microalgae as the treasure house of new drug development in the future. The research articles illustrate the diversity of marine microbial natural products and their biological activities and highlight the importance of developing new methods to encourage the discovery of new compounds.
